# A qualitative study on expectant and new parents’ perceptions of *Interplay*, a digital support tool for parents’ couple relationship and parenting

**DOI:** 10.1186/s12884-025-08485-0

**Published:** 2025-12-01

**Authors:** Angelica Holst, Frida Lygnegård, Henrik Engström, Margaretha Larsson, Rajna Knez, Caroline Bäckström

**Affiliations:** 1 Faculty of Caring Science, Work Life and Social Welfare, University of Borås, Borås, 501 90 Sweden; 2 Youth Guidance Centre, Regionhälsan, Region Västra Götaland, Falköping, 521 46 Sweden; 3 Department of Rehabilitation, CHILD, School of Health and Welfare, Jönköping University, Jönköping, 551 11 Sweden; 4 School of Informatics, University of Skövde, Post box 408, Skövde, 541 28 Sweden; 5 School of Health Sciences, University of Skövde, Post Box 408, Skövde, 541 28 Sweden; 6 Department of Psychiatry and Neurochemistry, University of Gothenburg, Gothenburg, Sweden; 7 Skaraborg Hospital, Skövde, Lövängsvägen, 541 42 Sweden; 8 Department of Caring Science, University of Borås, Borås, 501 90 Sweden

**Keywords:** Midwife, Antenatal care, Child healthcare, Professional support, Transition to parenthood, Relationships, Serious game

## Abstract

**Background:**

Parental transition means a change in the parents’ couple relationship as the baby becomes the focus, thereby affecting the parents’ communication and intimacy. Such a transition may either strengthen or strain the relationship. As becoming parents poses new challenges to the parental couple, the objective of this Swedish study was to explore how parents perceive Interplay, a digital support tool for their couple relationship and parenting.

**Methods:**

Swedish expectant and new parents were offered the digital support tool ‘Interplay’ (in Swedish: *Samspel*), which they played mutually with their partner for minimum two weeks. Semi-structured interviews were conducted with 12 expectant and 10 new parents, and the data were analysed using the phenomenographic method.

**Results:**

The results are presented in three descriptive categories: An opportunity to shed light on the couple’s relationship and shared parenting; Reflections about yourself and your partner; The design, sense of trust, interest and willingness to play.

**Conclusion:**

Interplay was perceived by the parents as supporting them in their couple relationships and shared parenting. The parents described that Interplay functioned as a catalyst for communication and reflection within the parenting couple, which broadened the parents’ perspective. Nevertheless, parents varied in their perceptions on whether Interplay was sufficiently developed to be implemented within healthcare and offered to parents. The current study contributes knowledge on how parents perceive that a digital tool, can be designed to support them in their couple relationship and parenting during the transition to parenthood.

**Trial registration:**

The research project is registered (02/10/2020) within the ISRCTN, with ID: ISRCTN18017741.

**Supplementary Information:**

The online version contains supplementary material available at 10.1186/s12884-025-08485-0.

## Background

Becoming a parent has been described as a major life-changing event in a person’s life [1], and the ability to adapt to these changes can be theorised within transition theory [2]. Transition is characterized by changes that occur over time and that affect a person’s identity, such as becoming a parent. The transition to parenthood is influenced by the person’s understanding and ability to cope with the changes that becoming a parent may entail. Furthermore, it may be understood that the transition is facilitated by knowledge, preparation and access to satisfactory social and professional support [2]. In theory, the parental transition is suggested to start during pregnancy and last up to 18 months after the child is born [3]. A meta-synthesis showed that parental transition may have both positive and negative impacts on parental couple relationships, strengthening or straining the relationship. The quality of the couple relationship is important for the wellbeing of both the parents and their child [4], and conflicts between parents may affect attachment security in early childhood (five years and under) [5]. According to a meta-analysis, marital satisfaction decreases in the first two years postpartum [6]. The ability to adapt to the changes that parenthood brings can be characterised by the individual’s development of understanding, self-confidence, and strategies to manage the situation [7], as well as the person’s wellbeing, emotional, physical [8], and social contextual circumstances [9] Therefore, care for parents and, specifically, professional support should address both parents’ preparation for the changes that parenthood brings, as well as their abilities to identify their own challenges and capacity to overcome such challenges [4]. Professional support in midwifery and child healthcare, for example, can be provided in several different ways, such as by guidance in parents’ use of digital support tools and could be considered *instrumental support*, which Langford et al. described as concrete help and support [10].

In recent years, it has become more common for parents to turn to digital tools for social support, information, and knowledge with questions concerning their parental transition. More parents are asking for extended digital solutions to support them in the changes posed by the parental transition to their couple relationship [11]. A review [12] showed that both parents and healthcare professionals experience challenges in evaluating the quality of existing apps, which may lead healthcare professionals to avoid recommending apps to parents. Among parents, commercial apps seem to be more popular than evidence-based apps, which have been shown to be more scientifically robust. This shows a need for more evidence-based apps, the design of which should include parents’ perspectives. For example, parents express that digital solutions should be developed in collaboration with healthcare professionals to be trustworthy, such as midwives who have knowledge about parents’ needs for support [12]. Previous studies have indicated that parenting apps often focus on learning about childbirth and infant care, neglecting learning about parents’ self-care and couple relationships. In the development of apps for expectant and new parents, there is, therefore, a need to shift the focus from supporting infant care to also strengthening parents’ self-confidence. Such apps should also support parents’ health in relation to their parenting and couple relationship [13].

An increasingly common development in healthcare is to support, teach, engage, and motivate people through digital game design elements developed to engage the user, increase motivation, and provide more effective, lasting, and entertaining experiences [14]. Previous research has shown, for example, that game elements can serve as a pleasurable way to promote reflection, communication and preparation for future parenthood in parenting preparation groups [15]. A genre in game development is serious games, which differ from pure entertainment games, as serious games intend to achieve specific goals, such as education, training, or health promotion. The goal of serious games is to engage players in a way that is both fun and effective in achieving the intended learning or behaviour change goals [16]. A previous review showed that serious games can be effective in promoting positive health outcomes. However, further research is needed to increase the understanding of the potential and limitations of specific game mechanics and design elements for different audiences [17].

In Sweden, *Interplay* (in Swedish: *Samspel*) was developed as a digital support tool for parents to use during parental transition. Interplay relates to parental couple relationships and parenting and is a serious game in the quiz game genre. Parenthood can be understood with the support of Family Systems Theory, whereby family members’ expectations, behaviours and feelings are mutually dependent on each other. For parents, a decisive factor for a healthy couple relationship can be the ability to handle the stressors that parenthood may bring [18]. Similarly, the transition can be understood to be facilitated by awareness and engagement [2], whereby game elements can promote interaction and thus also influence engagement and learning [19]. When using Interplay, expectant and new parents deal with questions concerning their couple relationship and parenting, and the tool is designed to be played by both parents in a couple relationship. When playing, the parental couple is encouraged to individually answer questions and thereafter mutually discuss and reflect on the questions raised within the game. Interplay is further described in the methods section. However, research is needed to explore whether Interplay functions as support from the parents’ perspective. Therefore, the current study aims to explore how parents perceive Interplay as a digital support tool for their couple relationship and parenting. In the present study, Interplay is described as a *digital support tool* that is available in an app format.

## Methods

This study originates from a research project with mixed methods, as described in the study protocol by [20]. The research project was retrospectively registered (02/10/2020) within the ISRCTN with ID: ISRCTN18017741, http://www.isrctn.com/ISRCTN18017741. The study protocol [20] describes four different studies (A–D), and the current study constitutes Study B, which focuses on parents’ perceptions of Interplay*.* In the present study, expectant and new parents used the digital support tool Interplay*,* which means that they played Interplay mutually with their partners. Interplay is further described below. The current study is a qualitative method with an inductive approach, in which the data has been analysed using a phenomenographic method [21]. Phenomenographic traditions believe that humans have access to the world through their experience, and that we cannot separate what is experienced from our perceptions. Human perceptions and knowledge of the world are thus not solely based on our senses but also dependent on our personal history of experiences. Phenomenography aims to explore these variations in perceptions, similarities, and differences [22].

### Interplay – a digital support tool

**Fig. 1:**
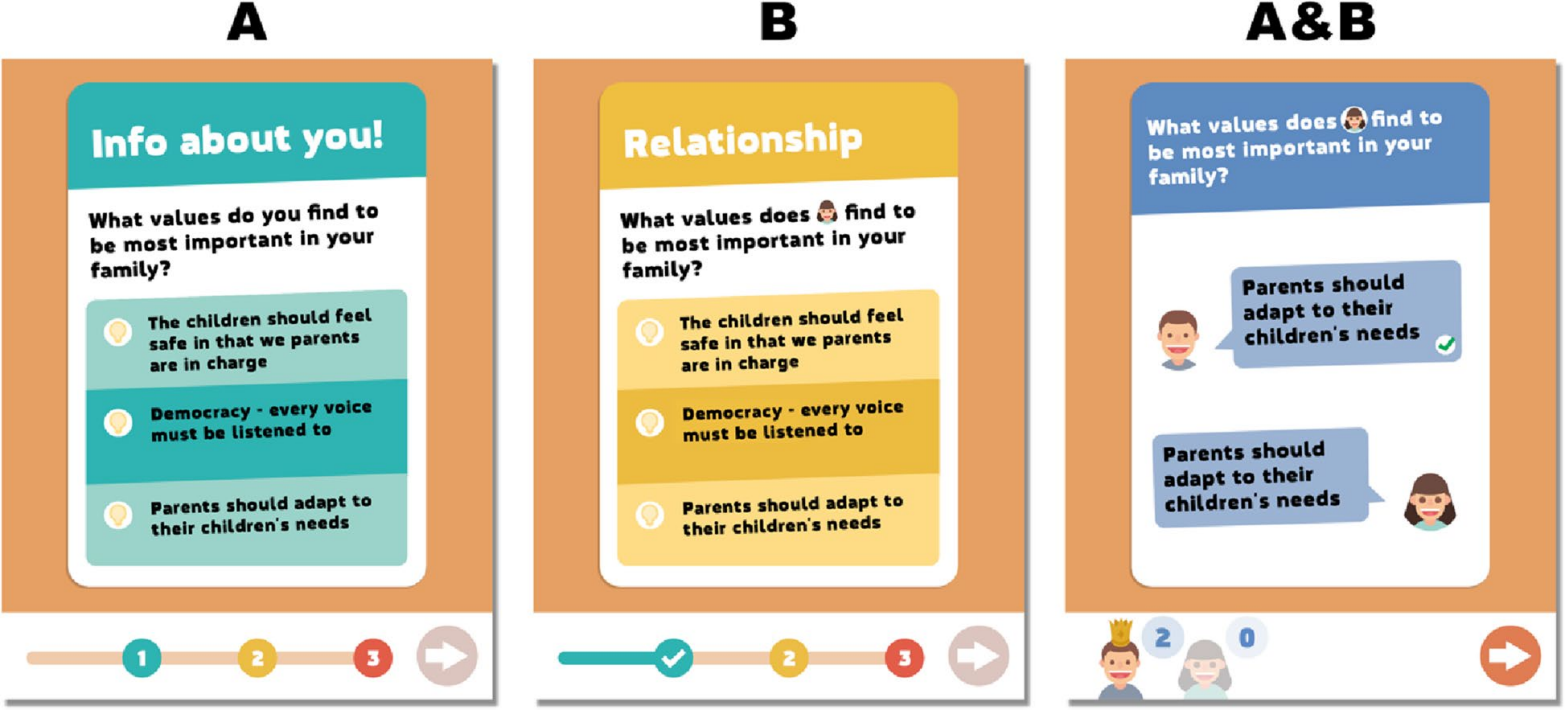
Screenshots of questions from Interplay (in translation from Swedish)

Interplay is a serious game in app format that includes concrete tasks and learning elements that aim to strengthen parents’ knowledge and ability to manage their roles as partners and parents. Interplay is played by a couple (Partner A and Partner B) and allows the player to adjust their own time for use by being able to play and paus among 15 quiz chapters at any time. The app contains no reminders or notifications, which means that players control their own usage. Each quiz chapter has between 10 and 17 questions, which each partner answers individually on their own device. Each quiz has three sections: in the first section, the player answers from their own perspective; in the second section, they answer from their partner’s perspective; and the third section presents trivia questions. The questions are matched so that if Partner A gets a specific question (Fig. 1, left) in the first section, Partner B will get the same question in the second section (Fig. 1, middle). In this way, the correct answer for Partner B to this question is given by Partner A and will be revealed when they get together to review the results (Fig. 1, right). Note that partner A is represented by a small picture in the phrasing of the question to Partner B.

When a chapter is completed by both parents, this becomes visible for them, whereupon the parents can choose a time and place to correct one or more finished chapters together. Partners A and B get access to the results by together placing their fingers on one of the smartphones screens at the same time, they can immediately see who was most correct in guessing what the partner answered as well as their responses to the trivia questions. The winner receives a price, such as telling their partner about their dreams for the future or a loving memory. Competition between two partners (parents) is thought to be a catalyst for social interactions between the partners (parents). The app format of Interplay enables parents to play regardless of time and space. The design has previously been well evaluated by players in the original Danish version (inn Danish: *Samspil*), although it has not yet been explored in any research study. For this study, the Danish version was translated into Swedish and revised to suit the Swedish context. The questions have thus been processed in line with the topics recommended in work with parental support within Swedish healthcare, such as family situation, care for the child, social situation, relationship between parents and access to support [23]. For more information about Interplay, please see the study protocol [20].

### Settings and participants

The present study was conducted in Sweden, which has approximately 10.5 million inhabitants [24]. Annually, a little more than 115,000 children are born in Sweden, which corresponds to approximately 220,000 new parents. To recruit parents, convenience sampling was performed, and social media such as Instagram and Facebook were used to advertise the opportunity for parents to participate in the study. In addition, parents were informed about the study through flyers at maternity units, as well as family centres within a rural–urban setting and a maternity unit that cares for approximately 2,700 births annually. In total, 28 parents in a couple relationship made contact, asking for further information about the study, they were informed about the reasons for the research, the approach and the background of the authors. Among them, 22 parents gave their consent to participate in the study and to complete their participation. The participants (12 mothers and 10 fathers) were between 27 and 39 years old; all of them were born in Sweden, and 20 had degrees from university. Among the participants, 12 were interviewed during pregnancy, between gestational weeks 16–40, 10 of the participants were interviewed after birth and had children between 2–14 months old. While 18 of the participants were first-time parents, 4 of the participants were expecting or had had their second or third child. All participants also had a partner who participated in this study, which means that 11 both hetero- and homosexual parental couples participated in the study.

### Data collection

Upon consenting to participate in the study, parents were given access to Interplay and instructions on how to download and connect with their partner in the app. Participants were also provided with contact information for the first author if they needed support in using the app; no participant requested additional support. Data collection was conducted at least two weeks later through semi-structured interviews. The participants were able to choose to be interviewed with their partner or individually. Offering participants the opportunity to choose can promote a sense of control, as individual interviews can provide space for personal experiences without the influence of partners, while interviews with both partners together can provide room for conflicting stories [25]. All participants in this study chose to be interviewed individually.

The semi-structured interviews were based on an interview guide [see Additional file 1]; the participants were given introductory questions, and the interviews then continued in accordance with the answers that emerged. The interviewer and the participant were thus able to work together to awaken the participant’s meta-consciousness. During the interview, the interviewer took notes to enable follow-up questions based on the participant’s narrative. Within the phenomenographic tradition, semi-structured interviews are preferable, with the interviewer bringing the participant back to what is in focus for reflection while simultaneously offering interpretations of what emerged in the narratives earlier during the interview [26]. The interview guide included both open-ended and follow-up questions, such as *Please tell me about your perceptions of Interplay. Which parts of Interplay did you appreciate more than others? Did you perceive Interplay as a support in your parenting or your couple relationship? Were there any aspects of Interplay that you experienced as difficult to understand?* The interview guide and techniques, such as Zoom end-to-end encrypted and Dictaphone, were tested in two individual pilot interviews before data collection began. The technical equipment functioned satisfactorily, and the pilot interviews showed that the interview questions were easy to understand and respond to. No changes were therefore made to the interview questions after the pilot interviews, which were also included in the data analysis. To enable the participants to have time to use Interplay, the interviews were held for at least two weeks after the participants had had access to it. The interviews were held digitally by the first author using a video link, which made it possible for the interviewer and the participants to see each other. After the first four interviews were transcribed, all authors read through the interviews, and mutual discussions were made to reflect on the interviewer’s interview technique and the relevance of the interview questions. No changes were made according to the interview questions, and the rest of the interviews were carried out between October 2022 and April 2023. Data collection was stopped when the authors agreed that all questions had been thoroughly explored and that nothing new emerged in subsequent interviews [27]. They were audio recorded, lasted between 13 and 66 min (mean 36 min), transcribed verbatim, and the transcripts corresponded to 197 A4 pages, a 1.15-spaced type.

### Data analysis

Data analysis followed the phenomenographic analysis process described by Sjöström and Dahlberg [21]. In the first step, *familiarisation,* the transcripts of the participants’ perceptions were read several times to become familiar with the text. In the second step, *compilation*, different perceptions were identified and marketed in the qualitative data analysis software NVivo 13. Through the third step, *condensation*, the perceptions were condensed into their meaning and essence for a clearer overview. Similar perceptions were placed together through the fourth step, *grouping*/*comparison* the categories were compared towards each other and with the transcriptions. After discussions among the authors, three descriptive categories and five perceptions were clearly visible (Table 1). Each individual category was given a preliminary designation to indicate the perception described. The categories were named in the sixth step, *naming*, with a descriptive tag based on the core of their meaning, distinguishing them from the other categories. All authors participated in mutual discussions repeatedly during the process for data analysis. The authors have varying levels of experience with qualitative methods and represent both male and female perspectives. The authors represent a range of professional backgrounds, including midwives, specialist nurse, an occupational therapist, a psychiatrist, and a professor in game development. One of the authors is a PhD-student, four are associate professors, and one is a professor.

**Table 1:** Overview of descriptive categories and perceptions

Descriptive categories	Perceptions
An opportunity to shed light on the couple’s relationship and shared parenting	Time together with opportunities to communicate with one’s partner
	Balanced conversations that reach a deeper level
	Raises sensitive topics in advance
Reflections about yourself and your partner	Reflections that increase understanding and strengthen the couple
	Further development to reach a broader and more diverse range of parents
The design, sense of trust, interest and willingness to play	

## Results

Data analysis of the parents’ perceptions of Interplay as a digital support tool for their couple relationship and parenting resulted in three descriptive categories: *An opportunity to shed light on the couple’s relationship and shared parenting; Reflections about yourself and your partner* and *The design, sense of trust, interest and willingness to play.* The descriptive categories with the associated perceptions are presented in an overview in Table 1. In what follows, the results are exemplified using the parents’ quotes (translated and slightly edited for clarity in English).

### An opportunity to shed light on the couple’s relationship and shared parenting

The parents perceived that Interplay provided them with support for making time for each other, which could otherwise be difficult in everyday life. They described how they started to talk with their partner in connection with the using the game. The various of topics introduced in Interplay served as a basis for balanced and deeper-level conversations between the parents. This, the parents described, facilitated their mutual understanding, security, and trust, which they perceived as fostering feelings of self-confidence and strengthened the relationship between the partners.

#### Time together with opportunities to communicate with one’s partner

The parents perceived that access to Interplay was valuable to make time for each other in the couple relationship, as it could otherwise be difficult in everyday life, and the couple relationship risks being forgotten during parenting. With Interplay and the opportunity to play together, the parent couple was motivated to take time for each other, which gave the opportunity to take a break from everyday life, and to pay attention to each other instead. One of the parents said, “… *we kind of got the opportunity to take time for each other… we would just kind of crash on the couch otherwise… but this gave a lot more than watching some boring series, I think*” (Informant 9).

Interplay was perceived as enabling a pause, which parents described as valuable and beneficial for coping with everyday life and maintaining focus on their couple relationships. A common perception among the parents was that they took the time to talk with their partners in a way that they would not have done without Interplay. This created an opportunity for them to focus and listen to each other. They described Interplay as a framework for dealing with concrete questions and discussing relevant topics both during the time they used Interplay and in the daily life they shared together.

*Now we really got the opportunity to talk about a lot of things, but also after Interplay, we got to talk, so we continued the discussions and the conversation, so it was a very good conversation opener.* (Informant 22)

#### Balanced conversations that reach a deeper level

The parents perceived that the variety of topics and questions in Interplay contributed to balanced conversations between the couple that reached a deeper level. The varying topics and the game elements in Interplay, with opportunities for the partners to give each other compliments, the couples’ conversations were perceived to become more balanced than before using Interplay. According to the parents, this could reduce the risk that one of the parents would perceive parts of the conversations as criticism from the other.

…*especially this that you are forced to put positive things into words...it has been very important to us that you do not wish each other any harm...that it is clear that both want to move forward and to something better...that you should have opportunities to say both positive and negative things to each other* (Informant 18)

Parents described that Interplay enabled them to take their couple’s conversations to a deeper level regarding issues that concerned their couple relationship, values, and joint future as parents. Parents who were expecting their first child expressed uncertainty about what changes parenthood would bring. Hence, they found it difficult to know what topics to talk about to prepare for their future lives with the baby. For these couples, Interplay provided support on relevant topics to talk about. However, they perceived it a bit difficult to imagine life with children and to answer questions concerning their lives after the child was born, for example, questions about who changes the diaper most often, or who usually puts the child to sleep. Parents with more than one child perceived that, in contrast to the everyday conversations that often tended to be about the children and everyday life, their conversations from using Interplay concerned their couple relationship on a deeper level instead. This was positive, according to the parents, since in everyday life, the parents perceived that questions about the couple relationship tended to fall into the shadows.

*...the more children you have... the children take up time, that’s how it is, so the time for each other becomes less and less...since the children comes first...*(Informant 17)

Parental couples who felt that they had good communication with each other even before they played Interplay perceived that the digital support tool gave them an opportunity to touch on deeper topics with more focus than they had done before. They also perceived that additional questions that may encourage communication at an even deeper level, such as ethical questions, would have enabled more valuable discussions between the couple. Thus, using Interplay would have added more to the couple relationship and joint parenting, according to the parents.

*...we had a lot of great conversations when we went through these answers...we were able to delve into certain things that we hadn’t had time to do yet and it became a good format for talking things over before what’s ahead...we gained an increased understanding of each other in some cases and in others more confirmation that we actually know each other well and know how to react and how to handle situations together...a very good format because it has had a positive impact* (Informant 11)

#### Raises sensitive topics in advance

The parents described Interplay as containing direct questions on sensitive topics that are typically not addressed in their everyday conversations within the couple. “*We would never have come to talk about these things if we hadn’t received the questions* [in Interplay]” (Informant 22).

For example, parents described that certain sensitive topics aroused emotions or made it clear that the parents had different perspectives on a situation. The fact that such sensitive topics came up in Interplay was particularly appreciated by the parents and was perceived as liberating because the parents themselves did not have to bring them up with their partner. Instead, Interplay was perceived to address the topic and thus constitute neutral ground and support for parents to discuss the topic. The parents admitted that it was easier for them to say to their partner “*shall we play*” instead of “*shall we talk*”*.* As the parents answered the questions on their own while using Interplay, they felt more prepared to discuss different topics with their partners afterwards. This was described as having a preventive function, since the couple could avoid raising topics that may potentially cause conflicts. Instead, they had the opportunity to discuss various topics in peace and quiet. It was perceived that it was easier for them to listen to each other, and created an awareness of how the other parent saw things, which was described as increasing the parents’ understanding of each other.

*I think it’s a more uncharged situation when you sit with an app like this...you’ve decided that now we’re going to do this and now we’re going to sit and discuss...you don’t have ‘the gun loaded’ as if it were going to come when you’re sitting at the dinner table and you’re tired...with a game like this I think you take your time in a different way to sit down and talk...I think you get a completely different understanding of each other and I think you become more thoughtful* (Informant 20)

This, the parents expressed, enabled new perspectives that reduced feelings of stress and strengthened the relationship. A recurring topic that was particularly appreciated in Interplay among new parents was to be intimate and have sex. The parents perceived it as a sensitive topic and something that they sometimes avoided talking about because it was experienced as intimate and bordered by fears, expectations, and bodily changes. Therefore, discussions about intimacy and sex tended to be overshadowed by what concerns parenting and everyday life. According to the parents, playing Interplay was supportive for them to express and put their feelings into words.

*If there is any area that ends up in the shadows, it’s really like the intimate parts when you have children… it’s great that it’s being forced out* [in Interplay]*, you could almost have more of it* [in Interplay]*…we are very communicative* [as a couple] *but that’s maybe an area where we haven’t been as communicative… so in that way it was very good that it* [intimate questions] *was forced, and probably be like that for many couples I think*… (Informant 15)

Some parents expressed the risk that mainly parental couples who feel that they have well-functioning communication would be attracted to using Interplay, as other couples may strive to avoid discussing topics they perceive as difficult or sensitive. This was opposed by parents who already received professional support (i.e. couple therapy) for their communication difficulties. These parents perceived that using Interplay had improved their communication skills with their partner.

### Reflections about yourself and your partner

The parents perceived that using Interplay facilitated reflections about themselves and their partner. Such reflections concerned thoughts about their own upbringing, role as a parent, parenting, and couple relationship. The reflections were perceived as valuable since they increased their understanding and strengthened their couple relationships and joint parenting.

#### Reflections that increase understanding and strengthen the couple

The parents perceived that Interplay facilitated reflections about themselves and about their partner. They expressed that through their use of Interplay, they were invited to share and listen to how they perceived each other within the couple relationship. They further described that this process enabled them to learn more about themselves and each other, thereby broadening their understanding of how they were perceived by their partner.

*You think that you should be perceived one way, but then when you talk about it* [with your partner]*, you realise, well, ok, you perceive me in a completely different way… it was important for… my self-awareness somehow.* (Informant 11)

The parents described that these reflections were not only aroused when using the game but also extended beyond use, which meant that they resumed topics of conversation on other times as well. The questions in Interplay were perceived to broaden the parents’ perspective on life, their expectations, the distribution of roles in parenting and couple relationships, routines, chores and finances. For example, the parents described that it prompted reflections on their balance and equality regarding parental leave or caring for sick children with work and career, as well as their division of household chores at home and their individual strengths in connection with their couple relationship and parenthood.

*...I was happy because she thinks we both take responsibility at home, while I feel like she takes all the responsibility…it was fun to hear that she thinks I also take responsibility, because I don’t always feel that way myself...you get to feel like you’re contributing something here at home...it makes you want to do it more...*(Informant 21)

These reflections and broadened understanding of themselves and each other, were described as promoting equality and, thereby, strengthen couple relationship. They perceived a broader understanding of strengths within the couple relationship, which they believed confirmed their couple relationship and created feelings of belonging and closeness to their partner. This was described as increasing their self-confidence and promoting feelings of security, as well as develop them as a couple. Furthermore, it was perceived to contribute to an increased sense of security in the parental role, as the couple relationship, according to the parents, forms the basis and influences how they handle joint parenthood. The parents believed that the reflections enabled mental preparation for possible situations that parenthood may entail. Whereupon the joint reflections within the couple relationship were perceived to enable the parents to pay attention to their differences, which was further considered to strengthen their ability to complement each other and become a team. Interplay was also described as encouraging reflections on their own childhood, which the parents expressed contributed to new insights and values ​​regarding their own parenting role. One parent said:

*I thought about how to be with the child… what we want to convey to our child, values, and the view of what is important… that was important to talk about, what do we really want to convey to our child and what do we bring from our different backgrounds, from our childhood…* (Informant 10)

The parents who already had children perceived that Interplay gave them the opportunity to show each other appreciation in their roles as parents. Furthermore, Interplay was considered to create opportunities for parents to pause and reflect on their experiences and thereby see how their parenting roles had developed over time. In this way, parents believed that their self-confidence and feelings of being connected in their parenting were strengthened, which was perceived to promote presence and stability in their parenting.

#### Further development to reach a broader and more diverse range of parents

The parents suggested that Interplay should be further developed and refined to reach a broader and more diverse range of parents. Parents perceived Interplay as both enjoyable and valuable, and believed it could benefit other parents by promoting communication, strengthening couple relationships, and enhancing confidence in the parenting role. Several parents told their friends about Interplay and recommended them to use it.

*It kind of gave rise to good discussions… we have more friends who are pregnant right now, who are also expecting their first child… we got a good basis* [from Interplay] *to be able to discuss and prepare for what lies ahead and therefore I think it would be good if more of our friends could also take part in it… we recommend this* [Interplay]*…* (Informant 11)

There were different opinions among parents, on the one hand a common suggestion among the parents was that Interplay could be introduced to parents during pregnancy by the midwife within antenatal care, as expectant parents are in a responsive phase to support and are more likely to trust Interplay if it was introduced to them by their healthcare professionals. There were also suggestions that the digital support tool could be introduced in other areas of care where parents are, such as child health care or in parent groups.

*…they* [healthcare] *wouldn’t recommend something that’s completely wrong...so you would definitely trust that a lot...I think that would be a great idea...then you also know that it reaches everyone* (Informant 13)

Some of the parents who wanted Interplay to be a natural part of antenatal care perceived that it would be positive if the health care professional followed up on the parents’ use of Interplay to ensure that everyday life does not get in the way of using the digital support tool and playing become forgotten. Through such follow-ups, parents perceived that the importance of communication within the couple relationship could be highlighted by the midwife. Furthermore, some of the parents suggested including Interplay in child healthcare, being introduced and followed up by child healthcare nurses. According to parents, Interplay could be helpful for healthcare professionals to identify parents’ needs for additional professional support regarding the impact of parenting on the parents’ couple relationship. Parents suggested that the digital support tool could be integrated into healthcare-based parent groups to facilitate shared experiences and broaden perspectives on couple relationships and parenthood.

*…you can always learn something from others...whether it’s good or bad...i think it’s helpful to talk to other people about concerns, fears, hopes and such...when you’re pregnant...the only ones who can relate to what it’s like right there and then...are other people who are pregnant* (Informant 14)

On the other hand, some parents did not perceive that Interplay would gain more trust if it was recommended by healthcare professionals, as some parents may feel forced to use it. Similarly, some parents felt that it would need to be developed further to be ready for use in healthcare, for example through more advanced design and a greater variety of questions. Likewise, someone perceived that the digital support tool could be recommended in healthcare, but that it would be preferable to use it on your own without follow-up in healthcare.

*It would be like sitting there and going through the answers together with her or him... for us it was probably easiest or best to do it as a couple, it wouldn’t have helped with a third part, or maybe it would have, I don’t know, but then it would have become more like therapy, someone who would kind of coach, that in itself isn’t bad... I think it’s more than enough to put it on the parents...*(Informant 12)

### The design, sense of trust, interest and willingness to play

The parents perceived trust in Interplay since it was developed by a university; they found it fun and easy to use, as the game’s design made it easy to get started and navigate. The app format and the design were highlighted by the parents as important for the game to function as a digital support tool for their couple relationships and parenting. Interplay’s app format was perceived as easily accessible and not particularly time-consuming for the parents. Further, the parents perceived it as positive that they could play both the individual and joint parts when and for how long it suited them.

*It was easy, and we went quickly to play…it was a short time to play but gave longer discussions, which was fun. (Informant* 8)

Most of the parents perceived that the questions did not always have an answer option that was completely consistent with their own thoughts and values about themselves or their partner; in those cases, the parents chose the answer that felt closest. The parents felt it was important to have the opportunity to choose an answer option that agreed with their own thoughts and values. However, they shared that not having a perfect answer as an option was not an obstacle, as they could explain their choices and their own thoughts with their partners when they compared their answers together.

An important aspect that promoted the parents’ willingness to continue playing Interplay was the joy of playing. Some game elements were perceived as especially fun, such as the possibility to choose their own avatar in the game as well as being able to correct the questions and get access to the answers, by simultaneously placing their fingers on one of the smartphones screens. Also particularly appreciated was the prize in each quiz, which according to the parents created opportunities to give and receive compliments as well as opportunities to freely tell their partner about their dreams for the future. According to the parents, the prizes made it easy and fun to talk to each other. There were also recurring perceptions that the competitive elements in Interplay were important for the enjoyment of the game and for the desire to continue playing. “… *as far as the points are concerned, yes, it’s a bit of a spur … this reward in the end was also a bit of a competitive moment … I also want to win at some point …*” (Informant 10)*.* However, some parents perceived it as less important and saw a risk of conflict if both parties were too competitive.

Particularly appreciated in Interplay were questions concerning the parents themselves, the couple relationship, and the joint future, with some parents preferring only questions concerning their couple relationship and parenting, while others also appreciated the trivia questions. The fact that Interplay was presented by a university was commonly perceived as strengthening the trustworthiness of the digital support tool, which meant that the parents did not reflect so much on the credibility of the trivia questions. Some parents perceived that the answers to the trivia questions agreed with their own values and thus felt reasonable, which they perceived strengthened their self-confidence. However, if the answers had not felt reasonable, some parents indicated that they would have sought more information. Furthermore, if they had accessed Interplay in any other context, they would have wanted source citations to increase the trustworthiness of the trivia question section.

## Discussion

In this study, we aimed to explore how parents perceived Interplay as a digital support tool for their couple relationship and parenting. By using Interplay, parental couples were given the opportunity to deal with issues related to their couple relationships and parenting. Current results show that parents perceived Interplay as a digital support tool that strengthened them as individuals and in their shared relationships and parenting. However, different opinions emerged about whether the digital support tool can be for everyone, some of the parents believed that it is not suitable for parents who have problems in their relationship while others believed that it could function as a support regardless of the quality of the relationship.

In the current study, both parents in a couple used the digital support tool, and they played together, which the parents described encouraged to mutually touch on their expectations and the distribution of roles. The parents perceived that Interplay strengthened their everyday ability to communicate with each other both when using Interplay and afterwards. Previous research confirms that it can be challenging for new parents to spend time together, as parenting tends to take up more space in everyday life [28]. The extended support that parents experience beyond the time of use of the digital support tool in this study may be seen as an important aspect of sustainability for digital support tools in healthcare, since previous research has shown that it can be a challenge to maintain the user’s interest in digital support over time [29]. However, further research is needed to explore longitudinal effects from parents’ use of Interplay. It is also important to consider, when interpreting our findings, that the parents in this study had used Interplay for just over two weeks prior to data collection, which represents a limited period of time. Nevertheless, the parents in this study participated as couples, and used the digital support tool as couple, which could be considered as a study strength. According to previous research, there is a need to include both parents to a greater extent in the provided support [30] and that most apps are primarily targeted at mothers [31]. Since the digital support tool Interplay includes both parents, it can be seen to meet the requested support that previous research has shown.

The current results revealed that parents perceived it easier for them to say to their partner “shall we play”, instead of “shall we talk”, which suggests that Interplay provided support for the parents to express their thoughts and feelings directly or indirectly to their partner. This is in line with previous research that has highlighted the importance of parents’ feelings and well-being in relation to the ability to cope with the changes that parenthood may bring [8]. The opportunity for the parental couples to touch on different topics raised in the digital support tool was supportive of them in the changes that parenthood entails, such as their roles and distribution of responsibilities. Studies have shown that the transition to parenthood can bring existential thoughts and feelings regarding the couple relationship [32] as well as difficulties for parent couples to find an even distribution and balance with their new roles as parents and everyday chores [33]. However, in this study, parents’ engagement and communication might have been enhanced if the questions in the digital support tool had been tailored differently for expectant parents and those who had already become parents.

In connection with the use of the digital support tool, reflections were raised among the parental couples about their roles as partners and parents, which increased their understanding of themselves and each other. This is consistent with previous research that highlights the interdependence of the couple relationship, in relation to parents’ expectations, behaviours and feelings [18]. This process was not created solely by the digital support tool but by the couple themselves during use and through the related conversations and reflections that arose. Thus, the parents took ownership of the process, perceiving it as strengthening their couple relationship. This can be understood as in line with previous research on the possibilities of games to promote engagement and learning [19]. The process made them perceive that their couple relationship was the basis for joint parenting, which contributed to their increased safety and self-confidence in their new roles as parents. Therefore, it might be suggested that Interplay functioned as a catalyst for mutual conversations among parental couples. However, including more questions addressing ethical dilemmas in everyday parenting might encourage parents to engage in deeper, more reflective dialogues. Hence parents’ communication affects how they handle challenges that the transition to parenthood may bring [34].

The results revealed that the digital support tool could attract parents to varying degrees depending on their interest in communication. Previous research has shown that parents should be supported by healthcare professionals in their communication during pregnancy [35] and that there is a need to focus more on the social and emotional changes associated with the transition to parenthood [36]. Similarly, parents request more information and support from healthcare professionals about the relationship changes that parenthood may bring [37]. The importance of focusing on the parents’ couple relationship has been confirmed by previous research that has shown that a healthy couple relationship can function as a predictor of good health [38, 39] and that the quality of the relationship affects the parents’ transition to parenthood [40, 41]. By meeting expectant parents’ needs for professional support, the transition to parenthood can be promoted, thereby reducing unrealistic expectations and stress that risk affecting couple relationships [34].

Although the parents expressed that Interplay required further development, it still, to some extent, addressed their needs for extended support for their couple relationship. We can only speculate that this, in the longer term, may have implications for the parents’ health. It is reasonable to assume that the current study illustrates how a digital support tool can be valuable for parents during the transition to parenthood. The results revealed nuances in how parents perceived the potential integration of Interplay into healthcare services and its provision by healthcare professionals. Some parents viewed the inclusion of Interplay in healthcare as beneficial, while other argued that further development was needed before it could be recommended to parents by healthcare providers. A detailed account of the adjustments considered necessary by the parents is presented in Additional file 2. Nevertheless, if Interplay were to be recommended within healthcare, professionals should be given the flexibility and opportunity to tailor its implementation to the individual needs and expectations of each couple. In this context, further exploration is needed to broaden knowledge on how Interplay could serve as a supportive tool to enhance couples’ mutual understanding and facilitate work on their relationship and co-parenting. Previous research has shown that parents prefer digital support tools that are recommended by reliable sources, such as healthcare professionals [42], developed through a collaboration between healthcare professionals, app developers, and parents, and containing information that is based on evidence [43]. In addition, previous research has shown a need for further research on how to best integrate hybrid health promotion interventions with healthcare professionals to promote parental health literacy [44].

In the current results, parents did not reflect so much on the credibility of the digital support tool; however, some parents perceived that the fact that it was presented by a university was considered to strengthen trustworthiness. However, the information raised in the trivia questions was not presented together with a source of reference. Some of the parents mentioned this as a potential development area, which suggests the need to support parents in their information seeking and to assimilate evidence-based and credible information under the condition of support. Professionals might also be facilitators of parent’s use of credible digital support tools that may facilitate their parental transition. This is confirmed by previous research that has shown that parents experience challenges in sorting and evaluating digital information that they are exposed to [45].

The parents in this study highlighted important prerequisites for digital support tools to function as support; they must be easy and fun to use. No participants made contact for technical support in connection with the game and the result showed that Interplay was perceived as easy to use. However, perceptions of development opportunities for Interplay emerged. For future research, it would be valuable to explore development opportunities as well as difficulties to a greater extent, since more perceptions may exist among other parents. Achieving a good balance between game enjoyment and educational goals can be challenging. Overweighting the game’s educational goals and educational nature may risk losing the player’s enjoyment of the game; the opposite holds true when the enjoyment goals are overweighted [46]. For good-quality serious games, both goals must therefore achieve a good balance [47], regardless of how serious the educational or pedagogical goals are, the game should be kept fun, as the enjoyment of the game is the very means to the goal [48]. From the current results, it is reasonable to conclude that the digital support tool, Interplay, met the need for gaming enjoyment among the parents. In future research, it would be valuable to include younger parents and young adults. Parents in the current study perceived that Interplay could also be valuable for other ages and, for example, be developed for young adults’ couple relationships. This is also confirmed by previous research that has shown that couple relationships among young adults affect their health and gender equality [49].

### Methodological considerations

At the beginning of recruitment for the current study, the parents were given the opportunity to choose whether they wished to be interviewed separately or together. The parents chose the time and place for the interview and whether they wanted the interview digitally or in a personal meeting. Although the parents were interviewed about a digital support tool that they used together, all parents chose to conduct the interviews individually. The interview situations were clearly characterised by the parents’ everyday lives. For example, one of the interviews was held digitally with a parent who was breastfeeding her newborn child and surrounded by older children in their home. Another interview was held digitally as the parent ran errands in the construction trade. This reflects the challenges parents face in everyday life: the challenge of getting the puzzle of life to fit together [9].

In qualitative research, data collection is stopped when all questions have been thoroughly explored, and nothing new emerges in subsequent interviews. This process can be influenced by the interviewer’s experience with interview situations; an experienced researcher can produce very relevant information through interviews with a small group of informants, while an inexperienced interviewer may need more informants and interview opportunities [27]. In the current study, 22 parents were interviewed until the authors considered that no new perceptions had emerged. The analysis process and the development of the preliminary categories in the current study were carried out by the first author and discussed continuously with the other authors. To support the relevance of the categories, excerpts from the interviews were made visible in the form of quotes. Within phenomenography, the issue of credibility involves the relationship between the empirical and the categories to describe similarities and differences in experiencing a certain phenomenon [21] as credibility in qualitative research contributes to trustworthiness [50]. The participants consisted of both expectant and new parents, ranging in age from 27–39, and living in both heterosexual and same-sex relationships, which demonstrates variation between participants. This refers to transferability to similar contexts [50].

However, it is important to consider limitations of the study. First, the study only reflects the results of a specific tool that is available in Sweden and perceptions from parents born in Sweden. It can therefore be understood that further research is necessary to create an understanding of how parents’ diverse cultures may influence their perceptions, as well as to understand Interplay from an international perspective. Since norms and expected behaviour may differ among different cultures in relation to parenting [51]. Second, all expectant and new parents who showed interest in participating in the study were included, which means that inclusion was not based on how participants perceived their couple relationship or on their interest in developing it prior to gaining access to Interplay. This could mean that only parents who perceive a good quality in their relationship and an interest in developing have chosen to participate. Further research is therefore required to investigate how parents with varied quality in their relationship would perceive Interplay. Parents were given access to Interplay at least two weeks before the interview, which means that more research over time is needed to explore parents’ perceptions in the longer term. Similarly, it should be taken into account in connection with transferability that it is possible to find additional perceptions because people have different ways of perceiving phenomena [50].

Current study aimed to include expectant and new parents with all levels of education. However, most parents who chose to participate were highly educated. For transferability, more research is therefore needed to better understand how parents with lower levels of education perceive digital support tools for their relationship and parenting. In relation to educational level, previous research has shown that highly educated partners may be freer to prioritise emotional connection with their partners due to a higher degree of financial and material security, while partners with less education may be exposed to more challenges outside of the relationship that need to be addressed and prioritised over intimacy-promoting activities, such as health problems, unemployment, and economic pressures [52]. Low-income couples also report higher levels of stress and lower levels of relationship satisfaction and relationship stability, and they have more serious problems than higher-income couples once they seek professional support [53]. However, this does not mean that well-educated parents do not experience challenges, as other research has shown that even highly educated parents experience lower levels of satisfaction in their relationship [54]. In order to reach a wider variety of participants, it can be understood that underrepresented groups may require a different approach to be reached. For example, through a more long-term recruitment process where researchers create local connections and relationships with communities and social groups [55]. Furthermore, it is important to support all types of parents’ couple relationships in accordance with United Nations agreement on Agenda 2030 and global goals to promote people’s health (*Goal 3: Good health and well-being*) and equality in general (*Goal 5: Gender equality*) [56]. The findings show that the digital support tool explored in the current study offers multiple aspects of sustainability. Nevertheless, this is a limited study on Swedish parents’ perceptions of a digital app developed for Swedish users, and further research is needed to expand knowledge on how this app could serve as a support for parents from a broader international perspective. Such research could, for instance, include randomized controlled trials to evaluate the longitudinal effects. Similarly, more research is needed to extend knowledge on how healthcare professionals perceive Interplay as a digital support tool for parents’ couple relationships and parenting.

## Conclusion

The digital support tool, Interplay, was perceived as supporting parents’ relationship and parenting. Parents expressed that the paus from everyday life when playing Interplay, created a room and space for the parental couples to together shed light on their relationship. However, it was not the digital support tool itself that was perceived to strengthen the couple’s relationship, but the couples themselves through the conversations and reflections that arose in connection with its use. Interplay was described as a catalyst for both communication and reflection within the parent couple, which was perceived as broadening perspectives and creating deeper dialogues. Nevertheless, parents varied in their perceptions on whether Interplay was sufficiently developed to be implemented within healthcare and offered to parents. The current study contributes knowledge on how parents perceive that a digital tool, can be designed to support them in their couple relationship and parenting during the transition to parenthood.

## Supplementary Information

Additional file 1. Interview guide.

Additional file 2. Descriptive categories and parents’ suggestions for improvement and further development, based on experiences from Interplay.

## Data Availability

No datasets were generated or analysed during the current study.
