# Supplementary material for: A qualitative study on expectant and new parents’ perceptions of Interplay, a digital support tool for parents’ couple relationship and parenting
Source: BMC Pregnancy Childbirth. 2025 Dec 1;25:1286. doi: 10.1186/s12884-025-08485-0 (PMC12667086; doi:10.1186/s12884-025-08485-0)
Supplement: Supplementary file 1 — Additional file 1. Interview guide. [file 12884_2025_8485_MOESM1_ESM.pdf]

Additional file 1.
(Translated from Swedish and slightly edited for clarity in English)

## Background questions:

1) How old are you?
2) How do you define yourself (as a woman, man or other)?
3) In which country were you born?
4) Where do you live?
5) Have you completed primary school, secondary school or university education?
6) How long have you and your partner been a couple?
7) How many children do you have?
8) What experience do you have playing digital games?

## Interview guide:

1) How were your experiences using Interplay?
2) Was it easy to understand Interplay? Tell me more.
3) Was there any part of Interplay that was difficult to understand? Tell me more.
4) Which parts of Interplay did you appreciate more than others? Which parts and why those particular parts?
5) How did you perceive the questions in the game?
6) How appealing did you perceive Interplay?
7) How credible did you perceive the information in Interplay?
8) Has Interplay affected your role as a parent? If yes, tell me how?
9) Has Interplay affected the relationship between you and your partner? If yes, tell me how?
10) Did you perceive Interplay as a support in your parenting or your couple relationship? If yes, tell me how?
11) What has Interplay meant to you?
12) How do you think that Interplay should be introduced/informed to parents?
13) How do you think about the midwife at the antenatal clinic or the district nurse at the child health care clinic talking about Interplay?
14) Would you like to have the opportunity to talk about your experience playing Interplay with the midwife at the antenatal clinic or the district nurse at the child health care clinic?
15) What other possibilities do you see with Interplay?

### Examples of follow-up questions:

How do you mean?

Can you tell me more?

Can you give an example?

How did it feel to you?
