# Supplementary material for: A qualitative study on expectant and new parents’ perceptions of Interplay, a digital support tool for parents’ couple relationship and parenting
Source: BMC Pregnancy Childbirth. 2025 Dec 1;25:1286. doi: 10.1186/s12884-025-08485-0 (PMC12667086; doi:10.1186/s12884-025-08485-0)
Supplement: Supplementary file 2 — Additional file 2. Descriptive categories and parents’ suggestions for improvement and further development, based on experiences from Interplay. [file 12884_2025_8485_MOESM2_ESM.pdf]

**Additional file 2.** Descriptive categories and parents' suggestions for improvement and further development, based on experiences from Interplay.	
**Descriptive categories**	**Parents' suggestions**
An opportunity to shed light on the couple's relationship and shared parenting	• More topics that are perceived as sensitive<br>• Include topics related to holidays
Reflections about yourself and your partner	Opportunities to develop a version of Interplay for;<br>• parents with children of older ages<br>• parents to play together with their teenager<br>• couple relationships with adult children who have moved away from home<br>• couple relationships without children<br>• a version of Interplay that can be included in couple therapy<br>• a version of Interplay for teen and young adult couple relationships<br>• a version of Interplay as a support for the individual in connection with various crises in life<br>• a version of Interplay as a support for the individual's health in relation to issues related to work<br>• versions of Interplay that can function as a support in School Education with students. In connection with health promotion work, for example in subjects such as bullying and drug prevention<br>• version of Interplay in the field of mental illness
The design, sense of trust, interest and willingness to play	• More extravagant graphics<br>• Adapt the questions according to the phase the parents are in, at the moment, release new chapters as the pregnancy progresses and the baby grows<br>• More ethical dilemmas<br>• Develop case situations where parents can take a stand on how they would act<br>• Develop a score overview that shows current standings and makes visible the couple's more and less strong areas<br>• Links for more information and support in various areas
